# Editorial: Mapping psychopathology with MRI and connectivity analysis

**DOI:** 10.3389/fnhum.2023.1141569

**Published:** 2023-02-01

**Authors:** Long-Biao Cui, Yongbin Wei, Min Cai, Hua-Ning Wang, Hong Yin, Baojuan Li

**Affiliations:** ^1^Department of Radiology, The Second Medical Center, Chinese PLA General Hospital, Beijing, China; ^2^Schizophrenia Imaging Laboratory, Fourth Military Medical University, Xi'an, China; ^3^Complex Traits Genetics Lab, Vrije Universiteit Amsterdam, Amsterdam, Netherlands; ^4^Department of Psychiatry, Xijing Hospital, Fourth Military Medical University, Xi'an, China; ^5^Department of Radiology, Xi'an People's Hospital, Xi'an, China; ^6^School of Biomedical Engineering, Fourth Military Medical University, Xi'an, China

**Keywords:** psychiatric disorders, neurological diseases, connectivity, mapping, magnetic resonance imaging

Different connections exist between different neurons and brain regions, constituting a complex and extensive brain network. Modern brain science research shows that the realization of many higher cognitive functions relies on the synergistic cooperation between different brain regions, not just on a specific brain region. The pathogenesis of many neurological and psychiatric disorders (e.g., schizophrenia, depression, etc.) is, to some extent, due to abnormalities in the connections between related brain regions. Brain connections at the macroscale can be classified into three types: structural connectivity, functional connectivity, and effective connectivity. Structural connectivity refers to the anatomical connections between brain regions. Functional connectivity uses signals recorded from different brain regions to calculate a certain index reflecting the strength of the relationship between brain regions. In contrast, effective connectivity is a causal and directional influence. In contrast to functional and structural connectivity, effective connectivity can establish causal relationships between the actions of different brain regions and may provide insights to explore psychopathology's neural mechanisms.

The present Frontiers Research Topic entitled “*Mapping psychopathology with MRI and connectivity analysis*” is part of the article collection series, “*Mapping psychopathology with fMRI and effective connectivity analysis*” (https://www.frontiersin.org/research-topics/3471/mapping-psychopathology-with-fmri-and-effective-connectivity-analysis). This Research Topic aims to introduce the use of magnetic resonance imaging (MRI) to investigate the neural mechanisms underlying neurological and psychiatric disorders (e.g., major depressive disorder, schizophrenia, Parkinson's disease, etc.). In addition, treatment effects were taken as the focus of the Research Topic, which included neuromodulation and psychotropic drugs, on the directional coupling between brain regions and whether alterations in connectivity persisted in subjects in remission.

At the macroscale, the human brain can be considered a complex and large network system consisting of structural and functional connections of different brain regions (Zhao et al.). Neuroimaging techniques have become a powerful method to study the structure, function, and metabolism of the brain in methamphetamine users (Yang et al.). Wang et al. enrolled diffusion weighted imaging (DWI) data of 42 adults with attention-deficit/hyperactivity disorder (ADHD) and 59 typically developing adults to explore the presence of abnormal connectomes in rich club structures in the brains of adults with ADHD, and the results showed that ADHD patients had reduced density of rich clubs in central structural nodes, mainly located in the insula, bilateral precuneus, left putamen, caudate nucleus, and right calcarine (Wang et al., 2021).

Functional MRI (fMRI) has emerged as an effective technique for the study of psychiatric disorders (Agoalikum et al.). Current neuroimaging findings suggest that major depression is not a dysfunction of a single brain region but associated with brain network dysfunction. An fMRI study of patients with major depressive disorder showed a significant decrease in the amplitude of low-frequency fluctuation and fractional amplitude of low-frequency fluctuation in the right precentral gyrus, a decrease in degree centrality in the left triangular part of the inferior frontal gyrus, and an increase in the left hippocampus after electroconvulsive therapy treatment (Li X. et al.). Agoalikum et al. used resting-state fMRI data to recognize disrupted brain connectivity differences among children, adolescents, and adults with ADHD. Their results showed that abnormal dynamic interactions and connectivity deficits were correlated with different groups and that these abnormalities differed between children, adolescents, and adults with ADHD. Li H. et al. studied changes in the functional connectivity of the vermis and brain regions in subjects with bipolar disorder at the resting state, and patients with bipolar disorder had reduced resting-state functional connectivity in the vermis and ventral prefrontal cortex compared to the HC group. A meta-analysis of patients with obsessive-compulsive disorder showed that the most consistent local connectivity abnormalities in obsessive-compulsive disorder patients occurred in the prefrontal cortex (Qing et al.). Gao et al. selected the striatum as a seed for functional connectivity analysis and found altered functional connectivity in the cortico-striatal network in patients with primary unilateral hemifacial spasm compared to healthy controls. In another study, functional connectivity changes in patients with vestibular migraine were assessed using resting-state functional connectivity analysis and compared with healthy controls. The results found that patients had reduced functional connectivity between the left inferior/middle temporal gyrus and supplementary motor area/the left superior frontal gyrus (Zhe et al.). Previous studies have confirmed that there are some changes in brain structure and function in patients with schizophrenia (Zhao et al.). Studies on local function in schizophrenia have found that cognitive dysfunction in schizophrenia is related to the function of the lentiform nucleus (Li P. et al.). Previous studies reported negative functional connectivity between the right lateral prefrontal cortex and the left putamen (Quide et al., 2013). One study found that quantitative and specific functional connectivity biomarkers may be valid radiomics signatures for individualized diagnosis of schizophrenia (Cui et al., 2018). In another study, Zhang Y. et al. found abnormal connectivity in brain language areas in patients with hallucinations.

However, the changes in the information flow, as measured by effective connectivity, of these distributed systems are still largely unknown. It has been shown that brain connectivity changes dynamically during the development of psychiatric disorders (Insel, 2010). Effective connectivity, as a type of brain connectivity, measures serve as promising biomarkers of schizophrenia (Li et al., 2017; Mastrovito et al., 2018). One study explored changes in causal connections between brain regions in adolescent-onset schizophrenia (AOS) patients and observed effective connectivity between the left superior temporal gyrus and the other four brain regions in the right hemisphere (superior frontal gyrus, angular gyrus, insula, and middle occipital gyrus) was impaired in patients with AOS. The results suggest that altered directional connectivity in the left superior temporal gyrus may have a significant effect on the development of AOS and as a possible biomarker for this disease (Lyu et al., 2021). Xi et al. obtained fMRI from first-degree relatives of patients with schizophrenia and found increased connectivity from the left anterior cingulate cortex to the right hippocampus and decreased connectivity from the right anterior cingulate cortex to the right hippocampus in patients' relatives compared to healthy controls (Xi et al., 2016). In another study, abnormalities in anterior cingulate cortex-related connections in the first schizophrenia *in vivo* were revealed by spectral dynamic causal modeling (Cui et al., 2015).

To summarize, this Research Topic highlights the application of MRI and connectivity analysis in the study of neurological and mental disorders. Furthermore, connectivity analysis using MRI data may provide a deeper understanding of the neural mechanisms of psychopathology.

## Author contributions

L-BC, YW, MC, H-NW, HY, and BL drafted and revised the manuscript. All authors contributed to the article and approved the submitted version.
